# The association of body size in early to mid-life with adult urinary 6-sulfatoxymelatonin levels among night shift health care workers

**DOI:** 10.1186/s12889-015-1770-x

**Published:** 2015-05-06

**Authors:** Cody A Ramin, Jennifer Massa, Lani R Wegrzyn, Susan B Brown, Jeffrey Pierre-Paul, Elizabeth E Devore, Susan E Hankinson, Eva S Schernhammer

**Affiliations:** Channing Division of Network Medicine, Department of Medicine, Brigham and Women’s Hospital and Harvard Medical School, 181 Longwood Avenue, Boston, MA 02115 USA; Department of Nutrition, Harvard School of Public Health, Boston, MA USA; Department of Epidemiology, Harvard School of Public Health, Boston, MA USA; Division of Biostatistics and Epidemiology, University of Massachusetts, Amherst, MA USA; Massachusetts College of Pharmacy and Health Sciences, Boston, MA USA; Applied Cancer Research – Institution for Translational Research Vienna (ACR-ITR VIEnna), Vienna, Austria

**Keywords:** Body mass index, Birth weight, Height, Somatotype, Melatonin, aMT6s

## Abstract

**Background:**

Adult body mass index (BMI) has been associated with urinary melatonin levels in humans; however, whether earlier-life body size is associated with melatonin, particularly among night shift workers, remains unknown.

**Methods:**

We evaluated associations of birth weight, body shape (or somatotype) at ages 5 and 10, BMI at age 18 and adulthood, weight change since age 18, waist circumference, waist to hip ratio, and height with creatinine-adjusted morning urinary melatonin (6-sulfatoxymelatonin, aMT6s) levels among 1,343 healthy women (aged 32–53 at urine collection, 1996–1999) in the Nurses’ Health Study (NHS) II cohort. Using multivariable linear regression, we computed least-square mean aMT6s levels across categories of body size, and evaluated whether these associations were modified by night shift work.

**Results:**

Adult BMI was inversely associated with aMT6s levels (mean aMT6s levels = 34 vs. 50 ng/mg creatinine, comparing adult BMI ≥30 vs. <20 kg/m^2^; P_trend_ <0.0001); however, other measures of body size were not related to aMT6s levels after accounting for adult BMI. Night shifts worked prior to urine collection, whether recent or cumulatively over time, did not modify the association between adult BMI and aMT6s levels (e.g., P_interaction_ = 0.72 for night shifts worked within two weeks of urine collection).

**Conclusions:**

Our results suggest that adult BMI, but not earlier measures of body size, is associated with urinary aMT6s levels in adulthood. These observations did not vary by night shift work status, and suggest that adult BMI may be an important mechanism by which melatonin levels are altered and subsequently influence chronic disease risk.

## Background

Melatonin (5-methoxytryptamine) is a hormone with cancer-protective properties [1], which can also enhance endothelial function [2,3] and reduce inflammation [4]. It is secreted predominantly during darkness (i.e., at night), with little production throughout the day [5]; however, its nocturnal production is also suppressed within minutes if light reaches specialized retinal photoreceptors at night (e.g., during night shift work) [6]. In the general population, measurements of melatonin’s primary urinary metabolite, 6-sulfatoxymelatonin (aMT6s), in first morning urine samples closely correlate with plasma melatonin levels measured during the previous night [7,8], and some epidemiologic studies have associated lower levels of aMT6s with an increased risk of breast cancer [9-11] and several cardiovascular disease markers[12-15]. In addition, body size throughout life (beginning at birth and including adult body mass index; BMI) has been related to breast cancer risk [16,17] and cardiovascular disease [18,19]. Moreover, inverse associations between adult BMI and aMT6s levels have previously been observed [14,20,21]. Still, previous studies have not examined the relation of earlier-life body size on melatonin levels in adulthood.

## Methods

The Nurses’ Health Study (NHS) II cohort was initiated in 1989, when 116,434 female registered nurses, aged 25 to 42, returned questionnaires on lifestyle, medical history, and health status. Biennial questionnaires are used to update this information, with >90% response rates for each questionnaire cycle. First morning urine collection occurred between 1996–1999. Collection methods and laboratory measurement of melatonin secretion have been described elsewhere [22-24]. Briefly, urine samples were assayed for aMT6s concentrations as part of previous nested case–control studies of breast cancer [9] and hypertension [23,25], with aMT6s levels creatinine standardized (aMT6s concentrations divided by concentration of creatinine) to account for differences in urine sample concentrations [20].

Information on body size was collected from questionnaires completed at urine collection and biennial cohort questionnaires. A woman’s birth weight and number of full-term pregnancies were ascertained in 1991. Women reported height, weight at age 18, and somatotype at ages 5 and 10 in 1989. To assess somatotype, women were asked to recall their body fatness at the specified age with a nine-level figure diagram [26], a method which correlates with weight and height measurements in childhood (r = 0.57 at age 5, r = 0.70 at age 10) [27]. In addition, women reported waist and hip circumference in 1993, and current weight was ascertained at urine collection and used to calculate adult BMI (kg/m^2^). Night shift work history was ascertained for the 2 weeks prior to urine collection (in number of nights worked), 2 years prior to urine collection (in months of night shift work), and cumulatively throughout life (in years) up to urine collection; our shift work assessments are detailed elsewhere [20,28]. aMT6s values that were below the limit of detection for the assay (<0.80 ng/mL, n = 10) were set equal to this limit. Because mean values of aMT6s concentrations differed by cycle at which aMT6s were measured in the breast cancer case–control study, we recalibrated aMT6s and creatinine values using drift samples. The original assay results and rerun results were highly correlated (r > 0.90) for all cycles, thus the different assays were measuring the same analyte despite differing absolute levels. Further details have been described elsewhere [24]. Absolute values of melatonin were similar in the breast cancer and hypertension nested case-control studies. In addition, we used the Generalized ESD Many-Outlier Procedure [29] to remove outliers in our aMT6s measurements from the breast (n = 7) and hypertension (n = 17) nested case-control studies. After these exclusions, there were 1,343 controls included in these analyses.

For our statistical analysis, we used the natural logarithms of urinary aMT6s measurements to improve normality of the outcome distribution, and estimated geometric mean levels of melatonin across categories of each exposure using linear regression. P-trends were calculated using continuous terms for our exposures. To reduce potential misclassification, we also calculated somatotype averaged over ages 5 and 10, to estimate childhood somatotype. Lastly, we stratified our analyses of body size and melatonin levels by median age at urine collection (<44 vs. ≥44 years) and night shift work, and used likelihood ratio tests to evaluate effect modification. All p-values were two-sided and p ≤ 0.05 was considered statistically significant. We used SAS Version 9.3 (SAS Institute, Cary, NC) for all analyses. This study was approved by the Institutional Review Board (IRB) of Brigham and Women’s Hospital (Boston, Massachusetts, U.S.).

## Results

There were modest differences in age and age-adjusted baseline characteristics by quartiles of aMT6s levels among the 1,343 women in this study (Table 1). In particular, women in the bottom quartile of aMT6s (median aMT6s, 20.4 ng/mg creatinine; 10-90th percentile, 9.2-27.6) were slightly older (mean age, 44.3 vs. 43.6 years), had higher BMI (mean BMI, 26.3 vs. 23.6 kg/m^2^) and greater pack-years of smoking (mean number of pack-years, 13.6 vs. 11.9), compared to women in the top quartile of aMT6s (median aMT6s, 84.4 ng/mg creatinine; 10-90th percentile, 67.6-124.2). In addition, 89% of all urine samples in the bottom quartile of aMT6s were first morning spot urine sample, compared to 98% in the top quartile.

**Table 1:** **Age and age-standardized characteristics at urine collection (1996–1999) of 1,343 women across quartiles of urinary aMT6s (ng/mg creatinine) in Nurses’ Health Study II** ^**a**^

**Characteristics**	**Quartiles of urinary aMT6s levels**			
	**Q1 (lowest)**	**Q2**	**Q3**	**Q4 (highest)**
N	335	336	336	336
Urinary aMT6s (ng/mg creatinine)^b,c^	20.4 (9.2-27.6)	37.0 (30.6-43.0)	53.0 (46.3-61.3)	84.4 (67.6-124.2)
Age (years)^c^	44.3 (4.5)	44.0 (4.2)	43.2 (4.4)	43.6 (4.1)
Birth weight, ≥ 7 lbs, %	64	62	59	63
Somatotype at age 5, ≥ diagram 5, %	7	7	8	5
Somatotype at age 10, ≥ diagram 5, %	11	10	10	10
Height (inches)	65.1 (2.6)	64.8 (2.5)	64.8 (2.6)	64.8 (2.4)
Body mass index at age 18 (kg/m^2^)	21.2 (2.9)	20.8 (2.5)	20.8 (2.9)	20.5 (2.3)
Current body mass index (kg/m^2^)	26.3 (6.0)	24.6 (4.7)	24.4 (4.5)	23.6 (4.0)
Weight change since age 18, ≥ 20 kg, %	26	17	17	11
Waist circumference (inches)	31.3 (5.0)	30.3 (4.2)	30.0 (4.5)	29.3 (3.6)
Waist to hip ratio	0.8 (0.1)	0.8 (0.1)	0.8 (0.1)	0.8 (0.1)
First morning urine sample, %	89	94	96	98
Full-term pregnancies, %	95	91	93	93
Physical activity (METs/week)^d^	19.6 (25.0)	20.3 (22.8)	20.5 (23.5)	20.0 (29.6)
Alcohol intake (g/day)	3.7 (6.2)	3.7 (6.7)	3.2 (5.6)	3.9 (8.2)
Pack-years of cigarette smoking	13.6 (11.8)	10.3 (8.3)	12.1 (11.1)	11.9 (10.3)
Current smoker, %	8	6	3	6
Nulliparous, %	18	18	18	20
Post-menopausal, %	11	8	6	9
Current post-menopausal hormone use, %	8	7	5	8
Ever oral contraceptive use, %	86	86	84	85
Current antidepressant use, %	10	14	14	12
Ever night shift work in 2 weeks prior to urine collection, %	9	11	7	7
Ever night shift work in 2 years prior to urine collection, %	15	14	14	10
Ever night shift work prior to urine collection, %	67	66	72	62

Values are means (SD) or percentages and are standardized to the age distribution of the study population unless otherwise noted.

^a^aMT6s, 6-sulfatoxymelatonin.

^b^Values are medians (10th-90th percentile).

^c^Value is not age adjusted.

^d^Metabolic equivalents from recreational and leisure time activities.

We observed a significant inverse association between adult BMI and aMT6s, suggesting higher levels of aMT6s measured in adulthood in women who were leaner at urine collection (P_trend_ = <0.0001). Specifically, women with BMI <20 kg/m^2^ had a mean aMT6s level of 50 ng/mg creatinine (95% CI, 45–56), compared to women with BMI ≥30 whose mean aMT6s level was 34 ng/mg creatinine (95% CI, 30–37) (Table 2). By contrast, after accounting for adult BMI, none of the other body size measures were significantly associated with adult levels of aMT6s (results also shown in Table 2). Further, when we averaged childhood somatotype, we observed no association with melatonin levels (data not shown).

**Table 2:** **Multivariable-adjusted geometric mean concentrations of urinary aMT6s (ng/mg creatinine) at urine collection (1996–1999) by categories of body size and night shift work among 1,343 women in Nurses’ Health Study II** ^**a**^

			**Model 1** ^**b**^		**Model 2** ^**c**^	
**Variable**	**Category definition**	**N**	**Geometric Mean (95% CI)**	**P-trend**	**Geometric Mean (95% CI)**	**P-trend**
Birth weight (pounds)^d^	<5.5	34	35 (29–43)		34 (28–42)	
	5.5-6.9	299	43 (40–46)		42 (39–45)	
	7.0-8.4	632	41 (40–44)		42 (40–44)	
	8.5-9.9	137	44 (40–49)		45 (40–50)	
	≥10	19	31 (24–41)	0.88	32 (24–42)	0.46
Somatotype at age 5	1 (leanest)	307	43 (40–46)		42 (39–45)	
	2	417	41 (39–44)		41 (38–43)	
	3	354	43 (40–46)		43 (41–46)	
	4	161	40 (37–44)		42 (38–46)	
	≥5 (heaviest)	86	41 (36–47)	0.47	44 (38–50)	0.50
Somatotype at age 10	1 (leanest)	254	41 (38–45)		40 (37–43)	
	2	426	43 (41–46)		42 (40–45)	
	3	326	42 (39–45)		42 (40–45)	
	4	193	41 (38–45)		42 (39–46)	
	≥5 (heaviest)	132	40 (36–45)	0.49	43 (39–48)	0.24
Height (inches)	≤62	236	42 (38–45)		42 (39–45)	
	63-64	391	43 (40–46)		43 (40–46)	
	65	193	43 (40–47)		43 (39–47)	
	66-67	316	40 (37–43)		40 (38–43)	
	≥68	207	41 (37–44)	0.30	41 (37–44)	0.23
Body mass index at age 18 (kg/m^2^)	<19	306	45 (42–48)		42 (39–46)	
	19-19.9	260	41 (38–44)		40 (37–43)	
	20-22.4	516	43 (41–46)		44 (41–46)	
	22.5-24.9	160	35 (32–39)		38 (34–42)	
	25-27.4	59	36 (31–42)		41 (34–48)	
	≥27.5	32	39 (31–49)	0.001	45 (36–56)	0.86
Weight change since age 18 (kg)	<5	452	45 (43–48)		41 (39–44)	
	5- < 20	633	42 (40–44)		42 (40–44)	
	≥20	229	36 (33–39)	<0.0001	43 (38–48)	0.66
Waist circumference (inches)	<26.75	163	45 (41–50)		41 (37–46)	
	26.75 - < 29	203	49 (45–54)		46 (42–51)	
	29- < 31	183	41 (37–45)		40 (36–44)	
	31- < 34.5	182	38 (34–41)		40 (36–44)	
	≥34.5	117	36 (32–40)	<0.0001	43 (37–50)	0.49
Waist to hip ratio	<0.72	150	46 (41–51)		44 (40–49)	
	0.72- < 0.75	162	44 (40–49)		42 (38–47)	
	0.75- < 0.79	220	42 (38–45)		41 (38–45)	
	0.79- < 0.82	154	38 (35–43)		40 (36–44)	
	≥0.82	158	40 (36–45)	0.02	43 (39–48)	0.55
Body mass index at urine collection (kg/m^2^)	<20	137	50 (45–56)			
	20-22.4	366	46 (43–49)			
	22.5-24.9	340	43 (40–46)			
	25-27.4	209	38 (35–42)			
	27.5-29.9	105	37 (33–41)			
	≥30	162	34 (30–37)	<0.0001		
Shift work 2 weeks prior to urine collection (nights)	0	1227	42 (41–44)			
	1-4	76	40 (35–46)			
	>4	37	38 (31–47)	0.29		
Shift work 2 years prior to urine collection (months)	0	1158	42 (41–44)			
	1-9	78	43 (37–49)			
	10-19	26	38 (30–49)			
	≥20	73	37 (32–43)	0.08		
Cumulative shift work prior to urine collection (years)^e^	0	447	43 (40–45)			
	1-9	804	41 (40–43)			
	≥10	85	42 (36–48)	0.37		

^a^aMT6s, 6-sulfatoxymelatonin.

^b^Analyses of body size adjusted for age at urine collection (5 year age categories), first-morning urine (yes, no), batch, number of pack-years smoked (0, <10, 10–24, ≥25 pack years), parity (nulliparous, 1–2 children, ≥3 children), physical activity in MET-hours/week (quintiles), and night shift work in 2 weeks prior to urine collection (0, 1–4 , >4 night shifts). Multivariable analyses for shift work adjust for the same factors except they adjust for body mass index (BMI) in kg/m^2^ at urine collection (<20, 20.0-22.4, 22.5-24.9, 25.0-27.4, 27.5-29.9, ≥30) instead of night shift work in 2 weeks prior to urine collection.

^c^Analyses of body size adjust for the same factors as model 1, plus BMI in kg/m^2^ at urine collection (<20, 20.0-22.4, 22.5-24.9, 25.0-27.4, 27.5-29.9, ≥30).

^d^Among women (n = 1,185) who were born full-term.

^e^Cumulative shift work, updating baseline lifetime shift work history through urine collection.

Results were similar when we restricted our analyses to non-smokers, first morning urine samples, or women reporting no night shift work in the two weeks prior to urine collection (data not shown). Moreover, night shift work was not significantly associated with mean levels of aMT6s in this sample regardless of whether we considered night shift work in two weeks, two years or cumulative night shifts over a woman’s lifetime prior to urine collection (P_trend_ = 0.29, 0.08, 0.37, respectively) (Table 2). Finally, associations of aMT6s levels with adult BMI (<25, 25–29.9, ≥30 kg/m^2^) did not significantly differ by shift work history (e.g., ever vs. never shift work in 2 weeks, 2 years, or cumulative night shift work prior to urine collection; P_interaction_ = 0.72, 0.07, 0.99, respectively) or age (data not shown).

## Discussion

Results from this study indicate that a higher adult BMI may adversely affect melatonin secretion, and night shift work did not appear to influence this observed association. Other measures of body size were not independently associated with aMT6s levels after accounting for adult BMI. Several studies have associated higher adult BMI with lower concentrations of aMT6s [20,21,30,31], although results have not always been consistent [32,33]. However, rodent models have provided substantial biologic evidence on the relation between decreased melatonin levels with obesity, weight gain [34,35,36] and metabolic syndrome [37,38]. Thus, our study suggests that adult BMI, not earlier-life body size, may influence an important mediator of the circadian system (i.e., melatonin) and later-life chronic disease risk. However, whether earlier life BMI (e.g., at age 18) mediates these effects cannot be ruled out completely, given the high correlation between BMI at age 18 and adult BMI.

Strengths of our study include a relatively large number of women with aMT6s measurements and a variety of information related to body size and potential confounding factors. Limitations include the use of a single aMT6s measurement which is susceptible to intra-person variation; however, first morning urinary aMT6s measurements remain fairly stable when measured repeatedly over several years (ICC = 0.72, 95% CI = 0.65-0.82) [22]. In addition, women were not asked if they worked the night shift within the past 24-hours prior to urine collection, which could have biased their first morning urinary aMT6s measure, yet results remained essentially unchanged when we excluded women with night shift work in two weeks prior to urine collection. Lastly, we cannot rule out potential misclassification of exposure covariates. For example, recall of self-reported somatotype at age 5 and 10 may be susceptible to misclassification; however, we averaged childhood somatotype to reduce potential misclassification and results were similar. Further, self-reported childhood somatotype recalled in later life correlates well with measured childhood body size [27], and earlier studies in our cohort have related important health outcomes with self-reported childhood somatotypes [39,40].

## Conclusion

In conclusion, our findings suggest that adult BMI is inversely associated with adult melatonin secretion, as assessed by first morning urinary aMT6s concentration, regardless of night shift work status. Additional large-scale prospective studies with more detailed and repeated assessments of melatonin are needed to further explore these associations.
